# Advancements in Artificial Intelligence for Medical Computer-Aided Diagnosis

**DOI:** 10.3390/diagnostics14121265

**Published:** 2024-06-15

**Authors:** Mugahed A. Al-antari

**Affiliations:** AISSLab, Department of Artificial Intelligence and Data Science, Daeyang AI Center, College of Software & Convergence Technology, Sejong University, Seoul 05006, Republic of Korea; en.mualshz@sejong.ac.kr

## Abstract

Rapid advancements in artificial intelligence (AI) and machine learning (ML) are currently transforming the field of diagnostics, enabling unprecedented accuracy and efficiency in disease detection, classification, and treatment planning. This Special Issue, entitled “Artificial Intelligence Advances for Medical Computer-Aided Diagnosis”, presents a curated collection of cutting-edge research that explores the integration of AI and ML technologies into various diagnostic modalities. The contributions presented here highlight innovative algorithms, models, and applications that pave the way for improved diagnostic capabilities across a range of medical fields, including radiology, pathology, genomics, and personalized medicine. By showcasing both theoretical advancements and practical implementations, this Special Issue aims to provide a comprehensive overview of current trends and future directions in AI-driven diagnostics, fostering further research and collaboration in this dynamic and impactful area of healthcare. We have published a total of 12 research articles in this Special Issue, all collected between March 2023 and December 2023, comprising 1 Editorial cover letter, 9 regular research articles, 1 review article, and 1 article categorized as “other”.

## 1. Introduction

The intersection of artificial intelligence (AI) and machine learning (ML) with medical diagnostics is rapidly evolving, heralding a new era of precision and efficiency in healthcare. This Special Issue of *Diagnostics* aims to capture the latest advancements and applications of AI and ML in the diagnostic process, spanning a diverse array of medical fields. AI and ML technologies are reshaping the landscape of diagnostics by providing tools that enhance the accuracy, speed, and predictive power of medical evaluations. These technologies have shown immense potential in areas such as radiology, pathology, genomics, and personalized medicine, where they are utilized for tasks ranging from image analysis and pattern recognition to the integration of vast datasets for individualized patient care. This Special Issue features contributions from leading researchers who explore various facets of AI and ML applications in diagnostics. The collected works encompass theoretical advancements, algorithm development, and practical implementations, demonstrating the broad impact and future potential of these technologies. The papers included in this Special Issue were gathered over a period spanning from March 2023 to December 2023, resulting in a diverse collection of high-quality research. We present a total of twelve articles: one Editorial cover letter, nine regular research articles, one review article, and one article categorized as “other”. Each contribution offers unique insights into how AI and ML can be leveraged to overcome current challenges in diagnostic medicine, enhance clinical decision-making, and ultimately improve patient outcomes. Through this Special Issue, we aim to provide a comprehensive overview of the state of the art in AI-driven diagnostics, highlighting both achievements and ongoing challenges in the field. We hope that this collection will serve as a valuable resource for researchers, clinicians, and technologists, fostering further innovation and collaboration in the quest to advance medical diagnostics. For more detailed insights, we encourage readers to explore each article and join the ongoing dialogue on the transformative potential of AI and ML in healthcare.

## 2. Overview of the Published Articles

This Special Issue on “Artificial Intelligence Advances for Medical Computer-Aided Diagnosis” presents a diverse collection of research that illustrates the significant impact of AI and ML on medical diagnostics. Below is a summary of each of the 12 articles published in this Special Issue. The Editorial cover letter, prepared by Mugahed A. Al-antar, who served as the Guest Editor (GE) of this Special Issue, provides an introduction to the Special Issue, outlining the scope of, the significance of, and the emerging trends in the integration of AI and ML in diagnostics. It sets the stage for the subsequent articles by emphasizing the transformative potential of these technologies in healthcare [1].

Following this, Gil-Rios et al. [2] introduce a new method for automatically classifying coronary stenosis using a feature selection technique. Their proposed method achieved a 99% discrimination rate using only four features, suggesting that this method could be useful in a clinical decision support system.

Ogunpola et al. [3] tackle the global health issue of cardiovascular diseases by focusing on improving early detection, particularly of myocardial infarction, using machine learning. Their study addresses the problem of imbalanced datasets, which can bias predictions, and employs seven classifiers: K-Nearest Neighbors, Support Vector Machine, Logistic Regression, Convolutional Neural Network, Gradient Boost, XGBoost, and Random Forest. The optimized XGBoost model achieved an outstanding performance with an accuracy of 98.50%, a precision of 99.14%, 98.29% recall, and an F1 score of 98.71%, significantly enhancing diagnostic accuracy for heart disease. 

Lee et al. [4] evaluate the diagnostic accuracy of two AI techniques, namely KARA-CXR and ChatGPT, in chest X-ray reading. Using 2000 chest X-ray images, their study assessed accuracy, false findings, location inaccuracies, count inaccuracies, and hallucinations. They found that KARA-CXR significantly outperformed ChatGPT regarding diagnostic accuracy (70.50% vs. 40.50%, according to one observer), false findings, and non-hallucination rate (75% vs. 38%). Both systems showed moderate inter-observer agreement. Their study highlights the superior performance of KARA-CXR in medical imaging diagnostics compared to ChatGPT. 

Marquez et al. [5] focused on using machine learning to differentiate between positive and negative influenza patients in Mexico, where influenza has been a persistent issue since 2009. They used a dataset of 15,480 records from 2010 to 2020, containing clinical and demographic data of patients tested with RT-qPCR. This study evaluated various classification methods and found that Random Forest and Bagging classifiers perform best in terms of accuracy, specificity, sensitivity, precision, F1 score, and the AUC. These methods show promise for aiding clinical diagnosis in settings where molecular tests are impractical. 

Ali et al. [6] investigated detecting Parkinson’s disease (PD) using voice attributes from both PD patients and healthy individuals. They employed filter feature selection to remove quasi-constant features and tested several classification models, including Decision Tree, Random Forest, and XGBoost models, on two datasets. Remarkable results were achieved on Dataset 1, with the Decision Tree and Random Forest methods achieving impressive accuracy. Ensemble learning methods (voting, stacking, and bagging) were applied to further enhance the results, and genetic selection was also tested for accuracy and precision. Their study found higher precision in predicting PD patients and better overall performance when using Dataset 1 compared to Dataset 2. 

Zakareya et al. [7] propose a new deep learning model for breast cancer classification, incorporating features like granular computing and attention mechanisms to achieve enhanced accuracy and reduce the workload of doctors. Their model’s effectiveness is demonstrated by its superior performance compared to other models on two case studies, achieving 93% and 95% accuracy on ultrasound and histopathology images, respectively.

Al-rimy et al. [8] examined the use of DenseNet169 for knee osteoarthritis detection using X-ray images. An adaptive early-stopping technique with gradual cross-entropy loss estimation is proposed to improve the model’s performance. This approach prevents overfitting and optimizes the number of training epochs. The proposed model demonstrates superior accuracy, precision, recall, and loss compared to existing solutions, indicating the effectiveness of adaptive early stopping and GCE for accurately detecting knee OA. 

Fatih Uysal [9] developed a hybrid artificial intelligence system to detect monkeypox in skin images. An open-source, multi-class image dataset was used, addressing data imbalance through augmentation and preprocessing. State-of-the-art deep learning models were employed for detection, and a unique hybrid model combining high-performing models with LSTM was created. The resulting system achieved 87% test accuracy and a 0.8222 Cohen’s kappa score. 

Al-Haidari et al. [10] propose a new deep learning framework for MR image reconstruction based on conditional Generative Adversarial Networks (CGANs) and U-Net. A hybrid spatial and k-space loss function is introduced to improve image quality by minimizing the L1 distance in both spatial and frequency domains. The proposed framework outperformed the traditional SENSE technique and individual U-Net/CGAN models in terms of the PSNR, while maintaining a comparable SSIM. Their CGAN-based framework also showed the best reconstruction performance, proving useful for practical cardiac image reconstruction by providing an enhanced image quality. 

Bhakar et al. [11] reviewed recent computational intelligence-based approaches for identifying disease severity levels. Their study focuses on Parkinson’s disease and diabetic retinopathy, but also briefly covers other diseases. This review examines the methodology, dataset used, and disease type of each approach, evaluating performance metrics such as accuracy and specificity. It also presents public repositories for further research in this field. 

Alnashwan et al. [12] conducted a systematic review to examine the latest advancements in artificial intelligence (AI) and computational intelligence for the detection and treatment of stuttering. They analyzed 14 journal articles from 2019 onward to investigate how AI can accurately determine and classify manifestations of stuttering, as well as how computational intelligence can contribute to developing innovative assessment tools and intervention strategies. Their review highlights the potential of AI and computational intelligence to revolutionize the assessment and treatment of stuttering, enabling personalized and effective approaches for improving the lives of people who have a stutter.

## 3. Limitations

Although the articles published in this Special Issue present valuable contributions to the field of medical diagnostics leveraging AI and machine learning (ML) techniques, some limitations can be identified across these articles:

(1) Many of these studies have specific focuses or use case scenarios, potentially limiting the generalizability of their findings to broader contexts or populations;
(2) Some articles may have methodological limitations, such as biases in data collection, imbalanced datasets, or reliance on specific classifiers, which could affect the reliability and applicability of their results;
(3) While many studies report impressive results in performance metrics such as accuracy, precision, recall, and F1 scores, the evaluation criteria and datasets used may not fully reflect real-world clinical scenarios, leading to potential overestimations of performance;
(4) The interpretation of results may vary across the studies, being potentially influenced by the researchers’ perspectives or biases, which could impact the conclusions drawn from the data.

## 4. Future Directions

Medical image diagnosis powered by artificial intelligence (AI) represents the cutting edge of improvements in healthcare. We should continually strive to enhance the health and wellbeing of our community by providing smart solutions that benefit both patients and physicians. AI technology has significantly advanced medical image diagnosis, improving accuracy, efficiency, and accessibility in healthcare scenarios. AI algorithms can analyze X-rays, CT scans, MRI scans, and patient records, such as those of patients with coronary stenosis or Parkinson’s disease, to detect abnormalities such as tumors, fractures, and infections. For example, AI can identify early-stage cancers that might be missed by the human eye. AI can interpret echocardiograms (ECGs) and cardiac MRI scans to detect heart conditions like arrhythmias, heart failure, and coronary artery disease. In light of this, this Special Issue highlights and disseminates the latest breakthroughs in AI-driven diagnostic technologies, with the aim of showcasing innovative research, fostering interdisciplinary collaboration, and exploring the transformative impact of AI on medical imaging and diagnosis. By bringing together contributions from leading experts, we hope to advance this field of research, improve diagnostic accuracy and efficiency, and ultimately enhance patient care and health outcomes.

The recent rise of large language models (LLMs) like Gemini and medGemini [13,14] or ChatGPT and BiomedGPT [15] presents a transformative opportunity for the healthcare industry, particularly in the realm of diagnosis. These powerful tools can significantly enhance existing diagnostic systems by providing more insightful explanations, ultimately leading to more accurate and efficient patient care. One key contribution of LLMs lies in their ability to generate detailed textual reports. By analyzing vast amounts of medical data, including patient history, test results, and relevant research, LLMs can produce comprehensive reports that not only outline a diagnosis but also explain the reasoning behind it. This detailed explanation assists healthcare professionals by offering a transparent and step-by-step breakdown of how the LLM arrived at its conclusions, fostering trust in the system and allowing doctors to leverage the LLM’s insights while incorporating their own medical expertise. While LLMs cannot directly generate heat maps (i.e., saliency maps) for medical images, they can play a significant role in the process. When integrated with computer vision models, LLMs are able to analyze medical images (X-rays, CT scans, MRIs, etc.), and they can be trained to detect and extract relevant features, anomalies, and patterns from these images. Through this, LLMs have the ability to generate textual descriptions of these highlighted areas that explain why they are important for a certain diagnosis. This provides a form of visual explanation alongside the LLM’s textual report. The combined power of detailed textual explanations and visual heat maps can empower healthcare professionals in several ways:

(1) It improves diagnostic accuracy by offering a comprehensive understanding of the LLM’s reasoning;
(2) It facilitates communication with patients by providing clear and accessible explanations of the diagnostic process [16,17];
(3) It fosters collaboration between humans and machines, allowing medical professionals to leverage the strengths of LLMs while retaining their own irreplaceable role in healthcare decision making [15].

However, it is crucial to acknowledge that LLMs are still under development, and their integration into healthcare systems requires careful consideration. Ensuring data privacy and security is paramount, and ongoing human oversight remains essential. In conclusion, LLM-based tools like Gemini and GPTs (Generative Pre-Trained Transformers) hold immense potential to revolutionize healthcare diagnostics. As mentioned above, the detailed textual explanations and visual heat maps provided by these tools can empower healthcare professionals by allowing them gain a deeper understanding of the diagnostic process, leading to improved accuracy and efficiency in patient care. As LLM technology continues to evolve, the future of healthcare diagnosis promises to be one of increased transparency, collaboration, and, ultimately, improved patient outcomes. The development of robust medical XAI models is often hindered by the limited availability of labeled datasets, particularly for rare diseases or specific imaging modalities. Generative AI techniques [18] offer promising solutions to address this challenge, enabling the creation of synthetic medical data across various modalities—text, images, signals, etc. LLMs like GPT-3 excel at generating synthetic patient records that closely mimic the structure, terminology, and statistical distributions of real clinical notes. Generative Adversarial Networks (GANs) have revolutionized medical image synthesis, producing realistic X-rays, CT scans, and MRI scans that can incorporate specific anatomical variations or pathologies. This not only increases the size of datasets but allows researchers to simulate diverse disease presentations. Additionally, GANs can augment datasets by introducing realistic variations to existing images or even by translating images between modalities (e.g., generating a potential CT scan from an MRI scan), reducing the reliance on acquiring data in multiple ways. Generative models are able to synthesize physiological signals like ECGs, EEGs, and others, replicating real-world patterns and anomalies. This can be leveraged to expand training data for diagnostic models and facilitate the development of monitoring devices. Furthermore, generative AI can create hybrid signals with controlled variations, simulating complex or unusual health conditions to diversify training data.

Finally, we can potentially leverage contrastive learning [19] to address the challenge of data-labeling exhaustion in the development of LLM-based tools similar to medGemini or BiomedGPT. Contrastive learning is a technique within self-supervised learning where a machine learning model learns by creating its own supervisory signals from unlabeled data. The primary goal of this is to train a model to recognize similarities and differences between data samples: the model learns an embedding space (a kind of representational map) where similar items are clustered together and dissimilar items are far apart. Meanwhile, deep active learning could prove to be capable of supporting and automating the process of labeling medical data.

## 5. Conclusions

In conclusion, this Special Issue underscores the transformative potential of artificial intelligence (AI) and machine learning (ML) in medical diagnostics, as evidenced by the collection of cutting-edge research it presents. While these advancements offer unprecedented accuracy and efficiency in disease detection and treatment planning across various medical fields, including radiology, pathology, genomics, and personalized medicine, several limitations persist. These limitations include the potential lack of generalizability, methodological constraints, and the need for the careful interpretation of results. Looking ahead, the integration of large language models (LLMs) like Gemini and medGemini holds promise for further revolutionizing healthcare diagnosis through empowering healthcare professionals with detailed textual explanations and visual heat maps to enhance the accuracy and efficiency of diagnosis. Despite these advancements, however, ongoing considerations for data privacy, security, and human oversight are essential. Ultimately, the future of healthcare diagnosis promises increased transparency, collaboration, and improved patient outcomes as AI technology continues to evolve and move forward to address current challenges.

## Abbreviations

AI	artificial intelligence;
XAI	explainable artificial intelligence;
ML	machine learning;
LLM	large language model;
LLMM	large language and multimodal model;
GAN	Generative Adversarial Network;
MRI	Magnetic Resonance Imaging;
CT	Computed Tomography.
